# Biophysical characterization of recombinant proteins: A key to higher structural genomics success

**DOI:** 10.1016/j.jsb.2010.05.005

**Published:** 2010-10

**Authors:** Masoud Vedadi, Cheryl H. Arrowsmith, Abdellah Allali-Hassani, Guillermo Senisterra, Gregory A. Wasney

**Affiliations:** a Structural Genomics Consortium, University of Toronto, 101 College Street, Room 839, MaRS Center, South Tower, Toronto, Ontario, M5G 1L7, Canada; b Campbell Family Cancer Research Institute, Ontario Cancer Institute, and Department of Medical Biophysics, University of Toronto

**Keywords:** Thermodenaturation, Protein stabilization, Ligand binding, Peptide array, Chemical probes

## Abstract

Hundreds of genomes have been successfully sequenced to date, and the data are publicly available. At the same time, the advances in large-scale expression and purification of recombinant proteins have paved the way for structural genomics efforts. Frequently, however, little is known about newly expressed proteins calling for large-scale protein characterization to better understand their biochemical roles and to enable structure–function relationship studies. In the Structural Genomics Consortium (SGC), we have established a platform to characterize large numbers of purified proteins. This includes screening for ligands, enzyme assays, peptide arrays and peptide displacement in a 384-well format. In this review, we describe this platform in more detail and report on how our approach significantly increases the success rate for structure determination. Coupled with high-resolution X-ray crystallography and structure-guided methods, this platform can also be used toward the development of chemical probes through screening families of proteins against a variety of chemical series and focused chemical libraries.

## 1 Introduction

With genome projects coming to completion almost a decade ago (Lander et al., 2001; McPherson et al., 2001; Venter et al., 2001; Waterston et al., 2002), and many similar breakthroughs in structure determination techniques and instrumentation, large-scale structure determination efforts were initiated (Burley et al., 1999; Christendat et al., 2000; Edwards et al., 2000; Gaasterland, 1998; Montelione and Anderson, 1999; Terwilliger et al., 1998; Vitkup et al., 2001). Such efforts led to the creation of many structural genomics groups that in time proved to be very efficient in high throughput protein structure determination (Edwards, 2009; Terwilliger et al., 2009). However, solving the structures of some proteins has been more challenging than others because of the diversity among proteins and their physical properties.

The production of proteins in a soluble form suitable for crystallization or nuclear magnetic resonance (NMR) studies has been a key element in structural genomics efforts. Attempting protein expression from different expression vectors or host cells and expression of domains or truncated proteins can significantly increase the probability of obtaining soluble and crystallizable proteins (Graslund et al., 2008; Lesley and Wilson, 2005; Vedadi et al., 2007). However, many proteins or domains remain unstable or partially soluble, often because they lack the appropriate cofactors or stabilizing ligands, or they are not in an optimum buffer condition. The presence of ligands often helps in obtaining well-diffracting crystals in such cases (Fedorov et al., 2007; Vedadi et al., 2006).

Nevertheless, structural genomics efforts have led to the deposition of thousands of structures, with an average of more than 150 structures per year from each of the major initiatives (Terwilliger et al., 2009). The functions of many of these proteins are either unknown or have been inferred from amino acid sequences. In the case of enzymes, little is often known about their substrate specificities. The identification of natural ligands (including peptide ligands) can help identify the native biochemical functions of proteins with unknown functions (Carver et al., 2005). High affinity, synthetic ligands can also serve as excellent research tools to investigate a protein’s function and specificity in a cellular context. Importantly, such efforts require an active, purified protein - a resource that is not trivial to produce, but is one that comprises the core of all structural genomics efforts. Ligand screening is a synergistic effort that facilitates both protein structural elucidation as well as the discovery of new functional information.

**Fig. 1:**
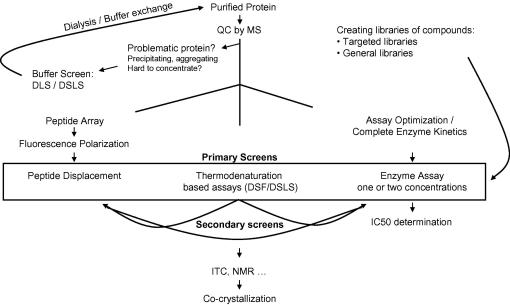
Characterization workflow in the Structural Genomics Consortium. All proteins are subject to quality control by mass spectroscopy. The sizes of purified proteins are confirmed in this step along with possible post-translational modifications (PTMs). The proteins that show signs of precipitation or aggregation and those that are difficult to concentrate are screened against a set of buffer conditions by dynamic light scattering (DLS) and differential scanning fluorimetry (DSF) or differential static light scattering (DSLS) to identify an optimum buffer condition that reduces aggregation. Enzyme assays are developed for each family of proteins (enzymes), and kinetic parameters are determined for each protein. An enzyme assay is the method of choice for screening enzymes when a proper assay has been developed. However, DSF/DSLS can be applied as a primary screening method for all proteins, regardless of their function. Peptide-binding proteins are screened against libraries of peptides immobilized on a cellulose membrane to search for substrates or interacting peptides. Fluorescence polarization is the method of choice for confirming peptide binding and, in turn, is used to screen for compounds that interrupt such peptide interactions (peptide displacement). Enzyme assays and peptide displacement can be used as secondary assays depending on the function of proteins. Isothermal titration calorimetry (ITC) and nuclear magnetic resonance (NMR) are low throughput methods to confirm binding when the number of tested compounds has been reduced by other methods. Co-crystallization is the final illustration of ligand binding; it provides binding site information and can help elucidate the structure–function relationship.

In the Structural Genomics Consortium (SGC, www.thesgc.org), we have established a high throughput platform using a 384-well format to characterize human proteins that includes screening the proteins against a variety of chemical series and focused chemical libraries, as well as investigating their binding and catalytic specificities. This platform includes thermodenaturation-based methods, including differential scanning fluorimetry (DSF) and differential static light scattering (DSLS), enzyme assays, an amplified luminescence proximity homogeneous assay (AlphaScreen), peptide arrays, a peptide displacement assay using fluorescence polarization, and dynamic light scattering (DLS) (Fig. 1).

Although there are other methods that can be used in the biophysical characterization of recombinant proteins, in this review we focus on the SGC platform and its application toward facilitating structure determination of large numbers of eukaryotic proteins, especially human protein families. We discuss its application, coupled with high-resolution X-ray crystallography and structure-guided methods, toward the development of chemical probes to proteins that are involved in a variety of post-translational modifications of histones, which in turn affect gene expression.

## 2 Screening for ligands using thermodenaturation-based techniques

Enzyme activity-based screening has long been used for high throughput compound screening. However, developing an enzyme assay for each protein is time consuming and may take several weeks or months. Alternative methods are also needed for non-enzyme targets. Many methods can detect ligand binding, but they may not be applicable for high or medium throughput screening. Isothermal titration calorimetry (ITC) is now widely used to detect protein interaction with compounds (Bains and Freire, 1991; Bundle and Sigurskjold, 1994; Chaires, 2008; Cooper and Johnson, 1994; Velazquez-Campoy et al., 2004; Velazquez Campoy and Freire, 2005). This method is reliable in determining K_D_ values for compounds, and it also provides a complete thermodynamic profile of ligand binding. However, it is a low throughput method and requires relatively high amounts of protein and ligand. Recently, miniaturized ITC units with reduced sample volumes (approximately 200 μl) have been developed, but these still require considerable amounts of protein, and each run may take about two hours to complete. Although not an option for screening high numbers of compounds, ITC is perhaps the most reliable technique for the quantitative measurement of ligand binding to a soluble protein.

Surface plasmon resonance (SPR) is another widely-used method for detecting protein–ligand interaction (Malmqvist, 1999; Rich and Myszka, 2000). This method uses biosensors to measure the change in the refractive index of the solvent near the surface of the sensor that occurs during complex formation or dissociation. The instruments are capable of characterizing binding reactions in real-time without labeling requirements. Consequently, SPR biosensors can be used to study the interactions of proteins and other biological systems. The method requires the immobilization of biomolecules to the surface of the sensor. The information obtained by SPR is both qualitative and quantitative, and it is suitable for measuring binding affinity (Karlsson, 2004). The advantage of SPR over ITC is that it requires less protein, and several samples can be processed simultaneously. The disadvantage is that one of the system components has to be immobilized on the surface of the sensor. ITC provides a direct measurement of the enthalpy of binding (ΔH), while in the case of SPR the binding constants have to be measured at several temperatures to obtain the enthalpy (Navratilova et al., 2007). Comparative studies show good correlations between the thermodynamic data obtained by SPR and ITC (Day et al., 2002; Navratilova et al., 2007).

**Fig. 2:**
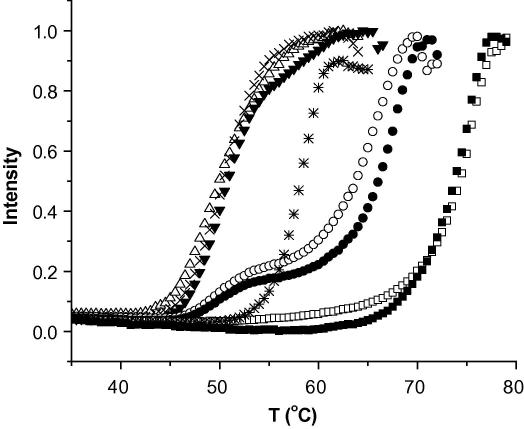
Detecting ligand binding using thermodenaturation. The effect of nucleotides on human GDP-D-mannose 4,6-dehydratase (GMD) was investigated by screening the protein both in the absence of any compound (Δ, control) and in the presence of 5 mM NAD^+^ (x), 5 mM NADH (▾), 1 and 5 mM NADP^+^ (○ and ● respectively), 1 and 5 mM NADPH (□ and ■ respectively), and 10 mM GDP (*). NADP^+^, NADPH and GDP stabilized GMD in a concentration-dependent manner, indicating that these compounds bind to GMD, whereas NAD^+^ and NADH did not have any effect (no binding). This is an example illustrating that the thermodenaturation methods can easily be used to investigate binding specificity of proteins. Abbreviations: GDP: guanosine diphosphate, NAD/NADH: nicotinamide adenine dinucleotide, NADP/NADPH: nicotinamide adenine dinucleotide phosphate.

The stabilization of proteins upon ligand binding is well documented in the literature (Brandts and Lin, 1990; Pace and McGranth, 1980; Schwarz, 1988). The coupling of the energies of ligand binding and protein unfolding results in ligand-dependent changes in the thermal stability of the protein–ligand complex relative to the stability of the protein alone (Brandts and Lin, 1990; Pantoliano et al., 2001). When the proteins are heated at a constant rate, it is possible to monitor unfolding and plot the signal as a function of temperature to obtain a sigmoidal curve representing the fraction of unfolded protein. The inflection point is usually referred to as the melting temperature (T_m_) where 50% of the protein is unfolded (Lo et al., 2004; Pantoliano et al., 2001). Upon ligand binding, the protein denatures at a higher temperature, and the difference in the T_m_ values in the presence and absence of the compound (ΔT_m_) reflects ligand binding. In the SGC, we use both fluorescence- (DSF) and aggregation-based (DSLS) thermodenaturation monitoring methods (Fig. 2) (Niesen et al., 2007; Senisterra et al., 2006; Vedadi et al., 2006). These are alternative ligand binding detection methods that are not dependent on protein function and can be employed for screening the majority of proteins in a 384-well format without the need for customization or assay development. Other methods, such as circular dichroism (CD) and differential scanning calorimetry (DSC), can also be used to assess protein thermodenaturation and ligand binding. However, both are low throughput methods.

Circular dichroism spectra provide an index of structure in proteins, and changes in the CD spectra may reflect perturbation of the structure. Therefore, folding and unfolding of a protein and the response to binding of a ligand can be monitored by CD spectroscopy. Heat-denaturation of proteins can be followed by monitoring the decrease in ellipticity at 220 nm as the temperature is increased, for example, from 25 to 85 °C (Chen et al., 1999; Epand and Epand, 2003; Narhi et al., 1999; Swint and Robertson, 1993). The pattern of the thermodenaturation profiles of proteins obtained by CD is similar to that obtained from DSLS and DSF. Profiling the stability of many proteins in parallel by CD and DSLS/DSF shows that these methods provide similar data (Allali-Hassani et al., 2009; Vedadi et al., 2006). Differential scanning calorimetry (DSC) is also useful for the study of heat-initiated phase changes in proteins and gives measurements of ΔH and other thermodynamic parameters of unfolding, ligand binding and complex formation. However, the throughput is low and it is not convenient for screening a large number of compounds. ATLAS™ (Any Target Ligand Affinity Screen) is another thermal denaturation based method that detects thermally unfolded and aggregated hexahistidine-tagged proteins. This method uses time-resolved fluorescence resonance energy transfer between two anti- hexahistidine antibodies, labeled with either a donor or acceptor fluorophore, that are simultaneously bound to the hexahistidine tags of aggregated protein (Patel et al., 2008; Thompson et al., 2008). Although this method is used in a 384-well format, it requires assay optimization for each protein (such as optimizing incubation temperature and time, and protein concentration) and some proteins with high T_m_ may not be amenable to this assay. Also presence of any aggregation in protein solution prior to heating increases the background and multiple heating and cooling cycles are needed to generate data points at different temperature.

**Fig. 3:**
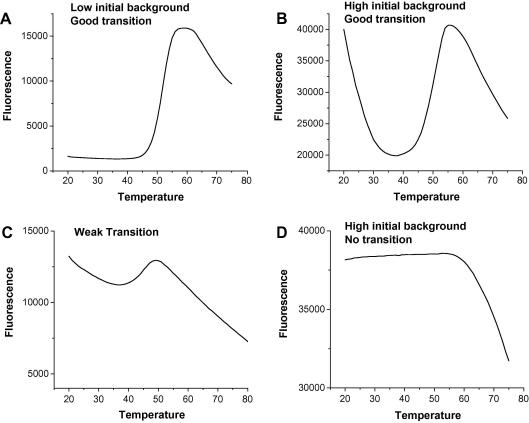
Examples of DSF profiles using SYPRO Orange as a reporter dye. The majority of proteins (>70%, (Vedadi et al. 2006)) are amenable to DSF using SYPRO Orange. Although many proteins may show perfect transitions with low fluorescence backgrounds (A), some may cause higher initial backgrounds with strong (B) or weak (C) transitions. Some proteins may not be amenable to DSF using SYPRO Orange due to high initial fluorescence backgrounds (D). Following the peak of intensity, a gradual decrease is observed, which is mainly due to the removal of protein from the solution due to aggregation and precipitation. A similar pattern can also be observed for thermodenaturation profiles obtained by DSLS.

**Fig. 4:**
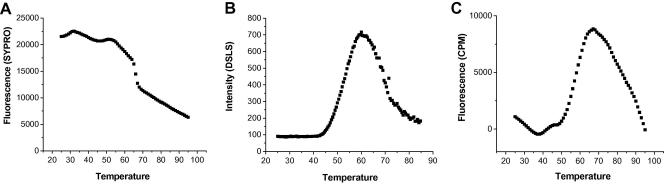
Thermodenaturation of pantothenate kinases (PANK3). Thermodenaturation of human PANK3 was monitored by DSF using SYPRO Orange (A), DSLS (B) and by DSF using CPM (C). Human PANK3 was not amenable to DSF using SYPRO Orange dye due to a high fluorescence background, and no transition was observed. However, in an aggregation-based assay (DSLS), a defined transition with a typical background was obtained. Replacing SYPRO Orange with CPM also produced a typical transition without significant fluorescence background interference.

In the differential scanning fluorimetry (DSF) method, external fluorescent probes are used to detect changes in protein surface hydrophobicity in a 384-well format. The fluorescence properties of these probes are quenched by water in aqueous solutions. However, in a hydrophobic environment introduced by protein unfolding during heat-denaturation, the fluorescence signal will increase (Epps et al., 2001; Slavik et al., 1982). The signal can be monitored using a variety of plate readers, including RT-PCR devices (Mx3005p™ from Stratagene, iCycler from Bio-Rad and LightCycler® 480 II from Roche) and fluorescence plate readers such as FluoDia T70 from PTI (Fedorov et al., 2007; Niesen et al. 2007; Vedadi et al., 2006). The first probe used for this purpose was 1-anilinonaphthalene-8-sulfonate (ANS), as reviewed previously (Slavik et al., 1982). Other probes with higher fluorescence quantum yields were later described (Epps et al., 2001). To date, the dye with the most favorable properties for DSF is SYPRO® orange, owing to its high signal-to-noise ratio (Niesen et al., 2007). Alexandrov et al., have recently reported the use of the thiol-specific fluorochrome N-[4-(7-diethylamino-4-methyl-3-coumarinyl)phenyl]maleimide (CPM) for stability profiling of membrane proteins (Alexandrov et al., 2008). The method uses the chemical reactivity of the native cysteines embedded in the protein interior as a sensor for the overall integrity of the folded state. Upon temperature-induced unfolding, CPM, which is nonfluorescent in the unbound form, reacts with the exposed cysteine residues, and an increase in its fluorescence intensity can be monitored. Some soluble proteins also have accessible hydrophobic areas and, therefore, their thermodenaturation cannot be monitored by using reporter dyes such as Sypro Orange that fluoresce in hydrophobic environments. In a case study, more than 25% of 60 soluble proteins examined by DSF using Sypro Orange were not amenable to such screening, partly due to a high fluorescence background (Fig. 3) (Vedadi et al., 2006). In comparison using CPM, the signal-to-noise ratio is higher for such proteins during thermodenaturation, and it may allow reliable T_m_ measurements (Fig. 4). However, the CPM method is only applicable to proteins with embedded cysteine residues. The analysis of PDB archive by Alexandrov et al., also showed that about a third of the membrane proteins do not have any embedded cysteine residues, and about 9% of the existing cysteine residues are not embedded in the protein interior. However, it might be possible to introduce cysteine residues by mutation without compromising the protein structural integrity to enable such measurements for ligand screening. They performed the assays successfully for several membrane proteins using a Cary Eclipse spectrofluorometer, which allows simultaneous analysis of four samples. We also employed this protocol in the SGC in a 96-well plate using the Mx3005p™ from Stratagene (ex; 350 nm, em: 516 nm) to assess the thermodenaturation of human pantothenate kinases (PANK3; Fig. 4) and obtained a typical thermodenaturation profile. Our results indicate that, although PANK3 is not amenable to DSF using Sypro Orange as the reporter dye due to a high fluorescence background, its thermodenaturation can easily be monitored by DSF using CPM or by differential static light scattering (Fig. 4). Alexandrov et al. also reported the limitations of the method, including the effect of pH on sensitivity and selectivity of CPM, as well as the need to eliminate reducing agents from purified proteins, and an increase in the fluorescence background if the protein contains exposed cysteine residues in unstructured loops. Similarly, CPM may react with the cysteine residues in the active site of enzymes and interfere with ligand binding. The authors also reported the application of CPM in generating isothermal denaturation profiles of several proteins. The application of high throughput isothermal denaturation in screening libraries of compounds using Sypro Orange (Senisterra et al., 2008) and detecting small stability differences between protein variants by DSLS has also been reported (Hong et al., 2007).

Another method to monitor protein denaturation is differential static light scattering (DSLS), a label-free method in which aggregation of unfolded proteins upon heat-denaturation is monitored by light scattering (Buchner et al., 1991; Senisterra et al., 2006). The intensity of the scattered light is presented as arbitrary values which, when plotted against the temperature, will result in plots similar to typical thermodenaturation profiles (Figs. 2 and 4). The inflection point of the sigmoid curve is called T_agg_ and is similar to the T_m_ obtained by DSF (Senisterra et al., 2006; Vedadi et al., 2006). DSLS measurements are performed using a StarGazer® instrument (Harbinger Biotechnology and Engineering Corporation) in 384-well clear bottom plates.

The majority of proteins are amenable to DSLS and DSF, and both methods provide comparable data (Vedadi et al., 2006). However, DSF cannot be used with hydrophobic proteins due to high fluorescence backgrounds. On the other hand, some proteins (especially very small proteins) do not aggregate over short time scales immediately upon denaturation and, therefore, may not be amenable to DSLS. The possibility of adapting a variety of fluorescence plate readers, such as RT-PCR machines that are readily available to researchers, and the ability to use commercially available software such as XLfit (IDBS) to analyze the data from 384-well runs, as well as the fact that such experiments can be performed on proteins regardless of their function, makes DSF an attractive 1st choice for protein characterization (Niesen et al., 2007). These experiments are typically performed in a 384-well format and each run, including the data analysis, takes less than two hours, thereby providing a screening capacity of more than 2000 data points per instrument per day. Screening soluble proteins for ligand binding is the primary application of DSF and DSLS. Recently, we also established that DSLS can also be used to screen membrane proteins (Senisterra et al., 2010). The specification and capabilities of DSLS and DSF and other methods of screening for ligand binding are compared in table 1.

Although ΔT_m_ or ΔT_agg_ values can be used for estimating the affinity of compounds for the majority of proteins, one should be careful in using these values to compare protein affinities for compounds with different physico-chemical properties (Holdgate and Ward 2005; Waldron and Murphy, 2003). The T_m_ or T_agg_ shift may not necessarily be the same for compounds with the same affinity, and it is dependent on the contributions of enthalpy and entropy to binding. Larger T_m_ shifts are observed for more entropically driven (e.g., hydrophobic) ligand binding. Also a range of different affinities, with different entropic and enthalpic components, might result in the same change in T_m_. There are cases in which a compound may bind tightly but no change in T_m_ would be detectable. For example, tight enthalpy-driven binding of a compound to the native state of a protein could potentially be masked by weaker, entropy-driven binding to the denatured state. It is even possible that the protein may exhibit a decrease in T_m_ in the presence of a ligand, even though the compound binds tightly to the native state (Holdgate and Ward 2005; Waldron and Murphy, 2003). In such cases, the use of other methods to detect binding, such as ITC, would be more informative. The use of enthalpy of binding as a tool for selecting compounds in lead discovery and aiding lead optimization has been proposed (Ladbury et al., 2010).

**Fig. 5:**
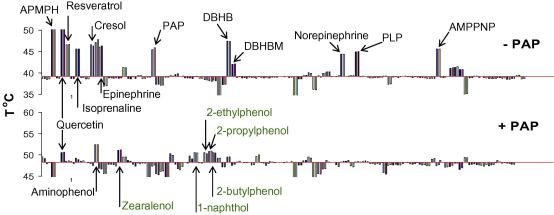
Protein chemical fingerprints. Chemical fingerprints of sulfotransferase 1C3 (SULT 1C3) in the presence (+PAP) and absence (-PAP) of 3′-phosphoadenosine 5′-phosphate (PAP) are presented. Cytosolic sulfotransferases (SULTs) comprise a family of enzymes that catalyze the transfer of a sulfonate group from 3′-phosphoadenosine 5′-phosphosulfate (PAPS) to an acceptor group of a variety of substrates. PAP is a product of the reaction. SULT 1C3 was screened in duplicate by DSLS against a library of 90 compounds both in the presence of a saturating concentration of PAP (10 mM) and in the absence of PAP. The horizontal line in each case marks the T_agg_/T_m_ of the protein, and each histogram indicates the effect of a particular compound on stability of the protein. Two histograms exist for each compound, representing the data in duplicate. The same compounds in the same order are represented in both horizontal lines. Each compound that was identified as a binder is indicated by the name of the compound in the figure. Abbreviations: APMPH: apomorphine, AMP-PNP: adenosine 5′-(β,γ-imido) triphosphate, PLP: pyridoxal 5’-phosphate, DBHB: 3,5-dibromo-4-hydroxy-benzoic acid (6,8-dichloro-4-oxo-4H-chromen-3-ylmethylene)-hydrazide, and DBHBM: 3,5-dibromo-4-hydroxy-benzoic acid (6-chloro-4-oxo-4H-chromen-3-ylmethylene)-hydrazide (Allali-Hassani et al. 2007).

A variety of chemical libraries are prepared for ligand screening in the SGC, some targeting specific families of proteins such as kinases (Fedorov et al., 2007) and sulfotransferases (Allali-Hassani et al., 2007), and some are designed to search for compounds with physiological relevance (Vedadi et al., 2006; Wishart et al., 2009). There are also commercially available libraries enriched for pharmacologically active compounds such as the Prestwick Chemical Library (www.prestwickchemical.com/index.php? pa = 26). Typically, a protein is screened against the libraries of compounds at a single concentration, and the “hits” are confirmed by repeating the thermodenaturation experiment in the presence of an increasing concentration of compound. Such screening can be employed to test a limited number of compounds in order to investigate the substrate specificity of an enzyme (Fig. 2) or to screen libraries with a large number of compounds. The resulting chemical fingerprints generated for different members of a family of proteins can be used to investigate binding specificity, active site similarity and selectivity of inhibitors (Allali-Hassani et al., 2007; Fedorov et al., 2007; Liu et al., 2009). The chemical fingerprint of an enzyme in the presence and absence of the co-factor can also be compared to obtain a more in-depth knowledge of the order of binding. These data provide preliminary information on the mechanism of inhibition (Fig. 5), and they are a convenient prescreen for more detailed enzymatic studies. Structure determination for more than 10% of the proteins solved by the SGC was only possible in the presence of ligands identified by such screening (Vedadi et al., 2006). Other methods, such as ITC, peptide displacement and enzyme assays, are routinely used in the SGC as secondary assays to confirm compounds identified by thermal denaturation methods.

In some cases, the proteins may not be amenable to DSF or DSLS, and we have therefore employed other screening methods such as the AlphaScreen™ assay (amplified luminescent proximity homogeneous assay) to test compounds and screen relevant chemical libraries (Wigle et al., 2010). AlphaScreen™ is a bead-based technology that was designed to measure the proximity of donor and acceptor beads conjugated to biomolecules of interest (Ullman et al., 1994). Alpha donor beads contain a photosensitizer (phthalocyanine), which converts ambient oxygen to an excited form of O_2_ (singlet oxygen) upon illumination at 680 nm. Within its 4-μsec half-life, singlet oxygen can diffuse approximately 200 nm in solution. This enables the excitation of the Alpha acceptor beads. AlphaScreen was originally designed as a direct homogeneous substitute for the ELISA immunoassay method where the coating, capture and wash steps are replaced by a pair of detection beads capable of recognizing two unique sites on the analyte of interest. As an example, the methylation of histone peptide substrates can be easily detected by specific methyllysine primary antibody-based interactions, in conjunction with streptavidin-coated donor and secondary antibody-coated acceptor AlphaScreen beads (Quinn 2010).

## 3 Screening for optimum buffer conditions

In large-scale protein expression and purification efforts, a preselected standard buffer condition is traditionally used for all proteins in the initial purification attempts. However, proteins have diverse physical properties, and their solubility and stability depend on the buffer conditions used during purification and storage. Considerable numbers of proteins show some degree of aggregation and precipitation during purification and storage (referred to here as problematic proteins). Frequently, these proteins are also difficult to concentrate. Altering the composition of the buffer used during purification can significantly influence protein stability. For example, the half-life of the DnaB protein from *Escherichia coli* was dramatically increased using an optimized buffer condition that was identified through screening a series of purification buffers (Arai et al., 1981). In some cases, it is crucial to express the protein in the presence of a ligand (co-factor, ion, etc.) to promote proper conformational changes or folding and to thereby increase solubility. For example, the expression of the recombinant human 11b-hydroxysteroid dehydrogenase type 1 in *E. coli* was increased by more than one order of magnitude in the presence of an inhibitor (Elleby et al., 2004).

In addition to screening for ligands, we adapted a combination of dynamic light scattering (DLS) and thermodenaturation-based screening at the SGC in order to find optimum buffer conditions that increase the solubility and stability of problematic proteins. DLS is very sensitive in detecting small particles in solution and can differentiate between non-aggregated proteins and proteins that form “soluble aggregates” or oligomers in solution. On the other hand, differential scanning fluorimetry (DSF) and differential static light scattering (DSLS) allow evaluation of the effect of buffer conditions on protein stability. Unlike DLS, DSLS is only sensitive to insoluble aggregates produced during protein precipitation and denaturation. The recombinant proteins may require several purification steps, which may take many hours. Furthermore, they may be stored for lengthy periods before being used and thus undergo a freeze–thaw process, and they are almost always subject to lengthy protocols such as crystallization screening and kinetic studies. It is therefore very helpful to select a buffer condition that makes the protein not only more soluble but also more stable. Conditions that make a protein more stable sometimes render improved solubility as well.

We previously reported that by using thermodenaturation methods to screen for stability, a buffer condition was identified in 50% of the cases that stabilized the protein by at least 4 °C compared with the original buffer (HEPES buffer, pH 7.5, 150 mM NaCl) (Vedadi et al., 2006). Most proteins were also stabilized in this assay by the addition of higher concentrations of NaCl. Only 27% of proteins were more stable at lower NaCl concentrations. In some instances, the identification of a stabilizing solution increased the ability to purify, concentrate or crystallize the protein (Vedadi et al., 2006). Using buffers and additives identified by DSF, Ericsson et al., reported a twofold increase in the number of crystallization leads compared with screening in the absence of the additives (Ericsson et al., 2006). Buffer optimization to improve purification yield and protein quality has also been reported (Mezzasalma et al., 2007).

**Fig. 6:**
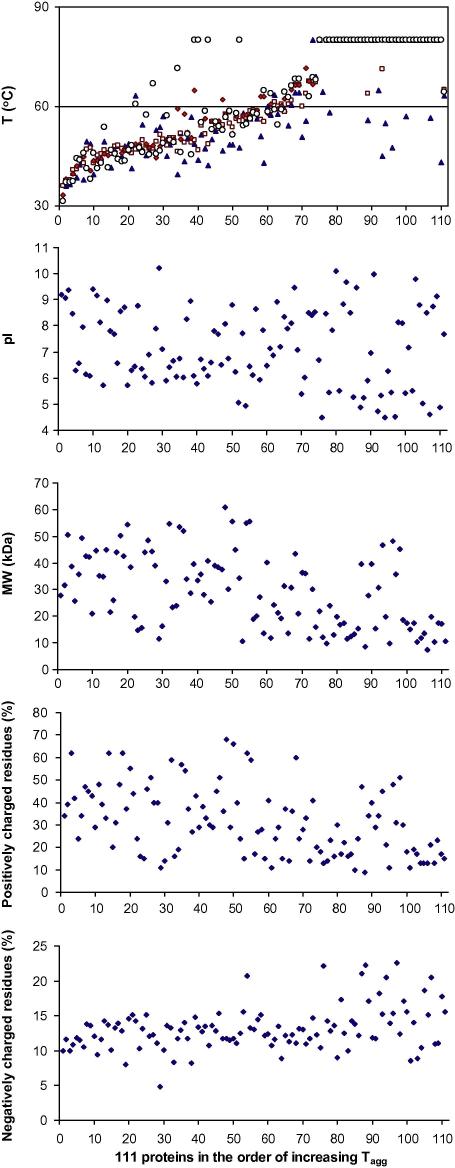
Mining the screening data. The proteins from malaria parasites were screened for stability by DSLS in buffers at pH 6 (▴), 7 (□), 8 (♦) and 9 (○) in order to find the optimum buffer condition for each protein. A higher stability and possibly better solubility in the optimum buffer condition is very helpful in long-term protein storage and when the protein is subject to lengthy processes such as crystallization or multi-step protein purifications. Many of these proteins showed significant changes in stability in different buffers, indicating the necessity of such a screening platform. Such large-scale screening also provides an opportunity to analyze the data in search for correlations between physico-chemical properties of proteins and optimum buffer conditions. This type of analysis could potentially help predict what buffer condition to use for each group of proteins based on their physico-chemical characteristics to reduce protein precipitation due to partial unfolding. Proteins are arbitrarily numbered from 1 to 111 and have been plotted in order from the least stable to the most stable protein. The isoelectric point (pI), molecular weight (MW), and percentages of positively and negatively charged residues are also plotted in the same order for the same proteins. Most proteins with no thermodenaturation transitions (top, right-hand side) appear to have lower molecular weights. Assuming that these proteins are not extremely stable in those conditions, the data suggest that very small proteins may not aggregate over short time scales immediately upon denaturation and may be less amenable to DSLS.

In search for optimum buffer conditions, more than 100 malarial proteins from different *Plasmodium* species were screened for stability by DSLS at various pHs (6, 7, 8 and 9). This provided an opportunity to seek possible correlations between protein stability related to the pH of a buffer and physical properties of these proteins (Fig. 6). About 25% of the proteins showed no thermodenaturation transition up to 80 °C in any buffer. Interestingly, a significant number of the remaining proteins (>50%) appeared to be most stable in an alkaline condition, versus only about 20% that were more stable in an acidic buffer (pH 6). The rest of the proteins were either most stable at neutral pH or showed no preference for any specific buffer. Although no correlation was observed between the isoelectric points of the proteins and their stability, there may be a weak correlation between their stability and their molecular weight and percentage of charged residues. The proteins that showed no transition in all or some buffer conditions were often small proteins (Fig. 6), raising the possibility that some very small proteins may not aggregate over short time scales and would therefore not be amenable to DSLS.

Dynamic light scattering (DLS) has long been used to identify optimal conditions for protein crystallization (Ferre-D’Amare and Burley 1994; Kadima et al., 1990; Moreno and Bolan˜os-Garcı´a 2000; Skouri et al., 1991; Zulauf 1992). To identify the optimum buffer conditions that reduce aggregation of problematic proteins, we routinely screen each protein against a set of 96 buffer conditions that include variations of pH, salt, and additives. Since we adapted this protocol for screening problematic proteins by DLS, analyzing the data to identify an optimum buffer condition from a high number of screened conditions and evaluating polydispersity or particle size distributions for each sample from the DLS screening output turned out to be difficult and time consuming. Alternatively, we took an unconventional approach to analyzing the data that helped us find optimum buffer conditions for proteins more quickly. We chose to focus on selecting conditions of lower scattered intensity as a measure of minimum aggregation (the intensity of scattered light is proportional to the square of the mass of the solute particle). The DLS instrument (DynaPro™, Wyatt Technologies) contains a feature that automatically shuts down the detector to protect it from damage when a sample is aggregated and the intensity of the light scattered is too high. The dynamics software that controls the data collection automatically adjusts the power of the laser to prevent loss of data due to this protection feature. Therefore, the higher the laser power and the lower the intensity, the more soluble the protein is in solution. The percentage of laser power and the beam intensity for all conditions can be quickly exported into Excel and ranked.

This approach has been used to identify better conditions to purify a variety of proteins. As an example, a variant of the sulfotransferase SULT2B1 (D191 N) was purified, concentrated and screened only after the optimum buffer condition reduced protein aggregation. The wild-type SULT2B1 protein is an easy-to-purify protein with a high yield in HEPES buffer (10 mM HEPES, 500 mM NaCl, pH 7.5), and it was concentrated to 18 mg/ml. Unlike the wild-type, the D191 N mutant that was purified under the same conditions precipitated during the concentration procedure. The protein was screened against 96 different buffer conditions by DLS after removing the precipitate. It was observed that in all conditions there was some degree of aggregation, and the laser power was lower than the expected 100% for the protein concentration employed (0.2 mg/ml). However, we found a condition (100 mM sodium citrate, 100 mM NaCl, pH 6.5) in which the intensity of the laser was the highest (65%). Such a protocol typically requires less than two hours to complete. Using this new condition, we were able to concentrate the protein to 10 mg/ml. Subsequently, it was possible to screen the protein by DSLS for ligand binding, which resulted in identification of ligands with stabilization effects of about 10 °C at 1 mM. It is worth mentioning that this approach is not used to decide what protein should or should not be put through the crystallization process. It is simply used to identify buffer conditions that keep the protein more soluble, providing an opportunity to further concentrate the protein, and enabling crystallization screening.

Size exclusion chromatography (SEC) is another method that can be used for estimating the sizes of macromolecules and the separation and quantitation of the non-covalent aggregate from the non-aggregated protein populations based on size (originally developed by (Porath and Flodin, 1959). This method is useful in assessing protein truncation, fragmentation and aggregation. It is an inexpensive technique with a potentially high sample throughput using a simple physical separation mechanism. In most cases, SEC works well, achieving good size separations and valid aggregation information. However, such results may require the validation of an orthogonal method to ensure that weakly associated aggregates are detected. Additionally, typical column-related problems may occur. The separation of non-covalent aggregation by a mechanism based solely on species size occurs only when there is no interaction between the solute and the column matrix. Although high performance SEC columns are designed to minimize non-specific interactions, most modern SEC columns are weakly anionic (negatively charged) and slightly hydrophobic, resulting in deviations from ideal size exclusion behavior (Chirino and Mire-Sluis, 2004; Clodfelter et al., 1999; Kamberi et al., 2004). The need to run many samples often results in the use of a mobile phase that is significantly different from the sample buffers. Changing mobile phase buffers for each protein sample would be impractical and time consuming. Therefore, SEC is less desirable for screening for optimum buffer conditions that reduce aggregation. Also, the need for upfront sample preparation to prevent column clogging (e.g., sample filtration) is not only time consuming, but it can also alter the amount and the size distribution of aggregation in the initial sample. Analytical ultracentrifugation (AUC) is another method that can accurately detect aggregates, and it is also sensitive to molecular shape (Berkowitz, 2006). This method has widely been used for determining the oligomerization state of proteins and estimating dissociation constants (Coutandin et al., 2009; Elkins et al., 2009; Pike et al., 2008), as well as for assessing the effect of buffers in increasing solubility and monodispersity of proteins (Bullock et al., 2009). However, AUC is time consuming and would be suitable for confirming data obtained from high throughput methods of screening.

## 4 Protein–protein interaction and peptide binding

### 4.1 Screening for binding peptides

The importance of protein–protein interactions has been widely recognized and investigated (Costa and Cesareni 2008). A major mechanism for protein–protein interactions is the binding of a globular protein or domain to a short peptide region of an interaction partner. Such protein-peptide interactions are highly enriched in signaling networks and are frequently mediated (positively or negatively) by post-translational modifications (PTMs), such as phosphorylation, acetylation and methylation (Pawson and Nash, 2003; Pawson et al., 2002; Santos-Rosa and Caldas, 2005; Seet et al., 2006). The generation of an appropriately modified binding partner for biochemical studies is usually best achieved by synthetic techniques.

Synthesizing and purifying peptides using the standard peptide synthesis technology as an alternative to full-length proteins for testing their substrate specificity are relatively slow and expensive processes. Several modified peptide synthesis procedures have been developed to address this problem (Frank, 2002; Geysen et al., 1984; Houghten, 1985; Pellois et al., 2002). Among these, the SPOT-synthesis technique is easy to handle and can be performed with minimum intervention (Frank, 2002; Kramer and Schneider-Mergener, 1998). This technique involves synthesizing and immobilizing peptides on a membrane which is suitable for mapping the protein–protein contact sites and profiling substrate specificity of enzymes (e.g., protein kinases, histone methyltransferases, etc.). The quantity and amino acid composition of the peptides can be easily modified, and detection of peptide–protein interactions involves simple blotting. The efficiency and quality of the synthesis of peptides on the membrane using standard L-amino acids have been validated by mass spectrometry (Frank, 2002; Hilpert et al., 2007). This method has been shown to be sensitive in detecting antibody-peptide interactions with K_D_ values as high as 1 mM (Kramer et al., 1999; Landgraf et al., 2004; Weiser et al., 2005). The ability to include modified amino acids in immobilized peptides provides an ideal tool for testing proteins that are involved in PTMs.

**Fig. 7:**
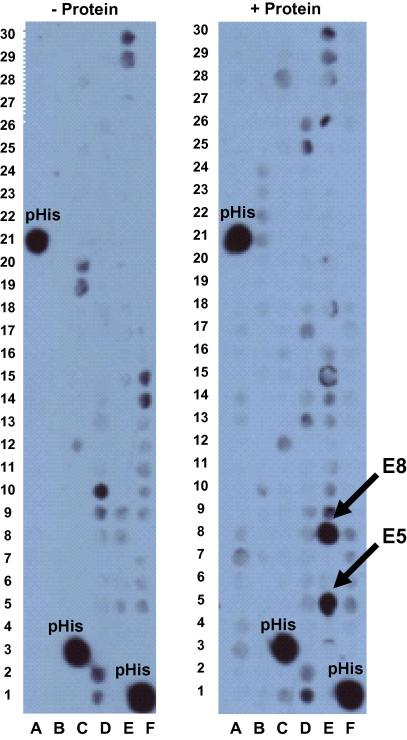
Screening for peptide-protein interactions using immobilized peptides on cellulose membranes. A total of 180 peptides were synthesized on a membrane. These peptides were designed based on an amino acid sequence of histone tails and included possible post-translational modifications such as acetylation and mono-, di-, and tri-methylation of lysines, and phosphorylation of serine and threonine residues. Histone methyltransferases (HMTs) and histone acetyltransferases (HATs) are responsible for methylation and acetylation of lysine residues on histone tails, which consequently affect chromatin condensation state and subsequent gene expression. In this experiment, a HMT was screened against this set of peptides, which were selected specifically for this methyltransferase, and poly-histidine peptides (12-mer) were also included as a positive control (pHis). The His-tagged protein binds to interacting peptides, and the presence of the protein can be detected by using anti-His antibodies (the HRP conjugate from Novagen was used in this experiment). All steps of the protocol were also performed in parallel without the addition of the enzyme as a control to eliminate false positives. Those spots that appeared on the membrane only when the protein was added (E5 and E8) were identified as hits (interacting peptides). Typically, the identified interacting peptides will be synthesized in solution, labeled with fluorescein, and their binding affinities will be further evaluated by fluorescence polarization.

In the SGC, we have established a peptide screening platform where proteins are screened against custom-made membranes with up to 600 immobilized peptides on each membrane (Nady et al., 2008). Desired peptides (8–20 residues long) are synthesized on and covalently linked to a modified cellulose membrane with a polyethylglycol linker using the MultiPep (Intavis) peptide synthesizer. This has the capacity to synthesize about 2400 peptides in four days. Immobilized peptides are then screened in an overlay assay to determine which peptides bind to the tested protein. The membrane is extensively blocked with skim milk, incubated with a His-tagged protein of interest, washed with phosphate buffered saline/Tween, and developed via western blot analysis. For example, screening a histone methyltransferase against a set of 180 peptides, shown in Figure 7, reveals that two specific peptides (E5 and E8) are clearly interacting with the protein. Performing the same experiment in the absence of the protein as a negative control indicates that the observed intensities for E5 and E8 are not artifacts. All three polyHis peptides that were used as positive controls were detected as hits with significant intensities (Fig. 7). However, some low intensity peptide spots associated with the experimental background are randomly present in both membranes. The amount of His-tagged protein used and the time of signal development may be optimized to reduce the background noise. The results of such screening are considered preliminary, and positive hits need to be validated by secondary assays such as fluorescence polarization.

The ability to incorporate modified residues (methylated, phosphorylated, acetylated, etc.) in any position within a peptide provides an opportunity to mimic post-translational modifications of proteins and to study the ways in which protein-peptide interactions are altered by these modifications. The peptide array approach is particularly useful for cases such as interactions with the natively disordered “histone tails” of nucleosomes, in which multiple sequences, each with a variety of modifications and combinations of PTMs, need to be screened for interactions with potential binding partners (Nady et al., 2008). Alternatively, other methods, such as the immobilization of glutathione S-transferase (GST) proteins and subsequent screening against fluorescently-labeled methylated histone peptides (Kim et al., 2006), or the use of biotinylated histone peptides for pull-down assays (Wysocka, 2006), have been used. However, these methods may not provide the same throughput.

### 4.2 Fluorescence polarization

Fluorescence polarization (FP) was first observed by Weigert in 1920 and later described by Perrin in 1926 (Collett and Schaefer, 2009). It is a well-established analytical technique that has been employed in a variety of biophysical characterization experiments, including the investigation of protein–protein, protein-peptide and protein-oligonucleotide interactions (Jameson and Seifried, 1999; LeTilly and Royer, 1993; Yu et al., 1996). FP-based immunoassay methods have also been used for clinical diagnostics (Bittman and Fischkoff, 1972; Chen and Potter 1992). FP-based screening has been adapted for high throughput screening (Du et al., 2007; Kawamoto et al., 2009; Reindl et al., 2009). An increase in the size of the labeled molecule (e.g., a peptide) through its binding to another macromolecule (in this case an interacting protein) affects its motion in solution, which can be detected by fluorescence polarization.

**Fig. 8:**
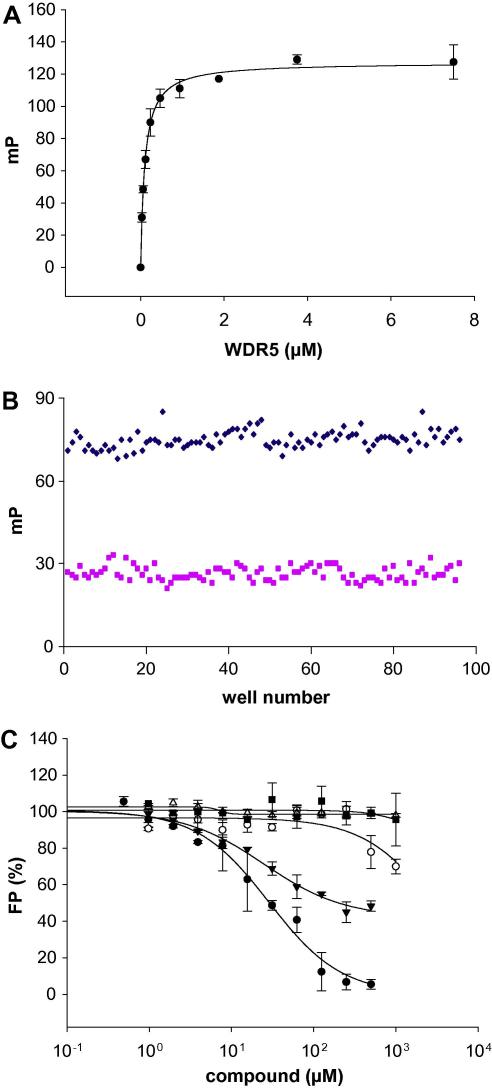
Application of fluorescence polarization in assessing peptide binding and screening for compounds. An 11-mer fluorescein-labeled peptide, similar to the first 11 residues of the C-terminal of the histone H3 that was dimethylated on lysine 4 (H3K4Me2) at 36 nM, was titrated with WDR5 from 30 nM to 30 μM. An increase in the observed fluorescence polarization (FP) signal was plotted against the WDR5 concentration (A). A K_D_ value of 100 ± 9 nM was calculated by fitting the data to a hyperbolic equation using SigmaPlot® 9. WDR5 (WD repeat domain 5), a WD-40 domain protein, is known to bind to the N-terminal of H3 when Lys4 is dimethylated (K4me2). This is a marker for further methylation by a methyltransferase MLL1 (mixed lineage leukemia-1). The 3D structure and mechanism of binding for this protein have been extensively studied (Couture et al. 2006; Han et al. 2006; Ruthenburg et al. 2006; Schuetz et al. 2006). Reproducibility of the FP signal and the possibility of peptide displacement were tested within a 384-well plate by repeating the experiment 100 times using the condition that produced the maximum FP signal (0.5 μM WDR5 and 36 nM fluorescein-labeled peptide; F-H3K4Me2) and the same number of positive controls (0.5 μM WDR5 + 36 nM F-H3K4Me2 displaced by 250 μM unlabeled H3K4Me2) (B). Excellent reproducibility was observed with a Z’-factor of 0.77, indicating that this methodology can be used for screening compounds. FP experiments were carried out in 100 mM potassium phosphate buffer (pH 8, 0.01% Triton X-100). In a peptide displacement experiment (C), the ability of a compound (▾) to displace the F-H3K4Me2 from the WDR5 binding site was confirmed. Similarly, a positive control (unlabeled H3K4Me2, ●) also displaced the labeled peptide. Monitoring of the change in FP signals due to peptide displacement is an efficient method of screening for compounds that interrupt protein–protein interactions.

We typically end-label the peptide with fluorescein, which has a fluorescence lifetime shorter than 10 ns, allowing the changes in polarized signal to be detected over a wider range of molecular masses (Pope et al., 1999). The observed value of FP depends on free and bound fractions of labeled molecules. Instruments such as the Synergy 2 Microplate Reader (BioTek) or ViewLux ultraHTS Microplate Imager (PerkinElmer) can be used for such FP experiments. Our collection of peptides is N-terminally labeled with fluorescein and purified. The binding assays are performed in a 10-μl volume at a constant labeled peptide concentration (usually around 40 nM) by titrating the protein within a range of low to high micromolar concentrations into appropriate buffer with 0.01% Triton X-100. The value of the measured FP is corrected by subtracting the value corresponding to the labeled peptide in the buffer (typically about 80 to 100 mP). To determine the approximate K_D_ values, the data are fitted to a hyperbolic function using SigmaPlot® software (Fig. 8A).

### 4.3 Screening for ligands using peptide displacement

In cases in which the peptides are known to bind to proteins, such as those interacting with histones (e.g., histone methyltransferases, acetyltransferases), the optimum FP signal can be used to screen for ligands. The compounds that bind to the same site as the binding peptide and can disrupt the protein-peptide interaction will typically displace the fluorescein-labeled peptide, resulting in a decrease in the initial FP signal. In the presence of all other compounds (no binding), the FP signal will remain unchanged. The effect of the compounds can be confirmed when the experiment is performed in the presence of different concentrations of compounds. This protocol can be applied in a 384-well format with a Z’-factor (Zhang et al., 1999) of 0.77 (Fig. 8B and C).

## 5 Other biophysical characterization methods

Nuclear magnetic resonance (NMR) is another biophysical method that can be used to study protein–ligand interactions (Baldwin and Kay, 2009; Coles et al., 2003; Skinner and Laurence, 2008). NMR can be used to evaluate the structural, thermodynamic and kinetic aspects of a binding reaction. Upon the interaction of a small molecule ligand with a protein target, a perturbation of the NMR spectrum occurs, and the detection of the perturbation is the basis of ligand binding analysis by NMR. The unique properties of small compounds and macromolecules allow selective detection of either the protein target or ligand. Within our platform, we use NMR screening as a secondary screen to confirm some ligand binding interactions (Fig. 1).

Many other biophysical techniques can also be very useful for characterizing individual proteins and, consequently, for aiding structural genomics efforts. However, a discussion of all these methods is beyond the scope of this review.

## 6 Concluding remarks

Over 35% of the protein structures deposited in the protein databank (PDB) by the SGC have been co-crystallized with small molecules that were identified through screening or were predicted to bind based on the expected function of the protein, and confirmed through characterization of the purified protein. In about 10% of all structures determined by the SGC, the determination of the protein structures was made possible only by the presence of the identified ligands (Vedadi et al., 2006). In addition, more than 50 structures have been determined in the presence of peptides selected through protein screening and characterization (Eryilmaz et al., 2009; Guo et al., 2009; Min et al., 2007; Schuetz et al., 2006). This platform has also been employed in a quest to identify chemical probes to explore the variety of ways in which proteins can be modified following translation. Such efforts have led to a rapid identification of the most potent inhibitor reported to date for the histone methyltransferase G9a (Liu et al., 2009) (PDB ID; 3K5 K). In addition, the components of this platform that were put together through the development of new large-scale characterization methods (Nady et al., 2008; Niesen et al. 2007; Senisterra et al., 2010; Senisterra et al., 2006; Senisterra et al., 2008; Vedadi et al., 2006) have helped in the characterization of large numbers of proteins (www.thesgc.org/publications/), including many variants of human proteins (Allali-Hassani et al., 2009; Hong et al., 2007).

## Figures and Tables

**Table 1:** Comparison of methods that assess ligand binding.

Ligand Screening Method	Throughput	Suitable instruments (examples)	Protein requirement	Advantages	Limitations
Differential scanning fluorimetry (DSF); SYPRO Orange	96-well format 96-well format 384-well format 384-well format	Mx3005p™ from Stratagene iCycler from Bio-Rad FluoDia T70 from PTI LightCycler® 480 II from Roche	<5 μg/well	– Independent of protein function – High throughput	– Sensitive to hydrophobicity of proteins (high fluorescence background) – Sensitive to compound fluorescence

Differential scanning fluorimetry (DSF); CPM	4⁎ (expandable) 96⁎⁎	Cary Eclipse spectrofluorometer Mx3005p™ from Stratagene	<5 μg/well	– Independent of protein function – Low fluorescence background – Applicable to membrane proteins – Potentially high throughput	– Limited to Cys-containing proteins – Solvent accessible Cys residues contribute to background – Sensitive to presence of reducing agent – effect of pH on sensitivity and selectivity of CPM – Sensitive to compound fluorescence

Differential static light scattering (DSLS)	384	StarGazer from Harbinger	<10 μg/well	– Independent of protein function – Label-free – Applicable to membrane proteins – High throughput	– Not applicable to reversible protein denaturation – Proteins that do not aggregate over short time scale immediately upon denaturation may not be amenable to DSLS

Surface plasmon resonance (SPR)	Relatively low	Biacore ProteOn XPR36; Bio-Rad	Low μg range	– Independent of protein function – Real-time on-and-off rates of macromolecule interactions – K_D_ determination for macromolecule interactions	– Requires protein or ligand immobilization – Relatively low throughput

Isothermal titration calorimetry (ITC)	Relatively low	MicroCal ITC	High μg to low mg range	– Independent of protein function – K_D_ determination for macromolecule interactions – Provides thermodynamic parameters	– High protein requirement – Relatively low throughput

Circular dichroism (CD)	Relatively low	JASCO Aviv Instruments	High μg to low mg range	– Independent of protein function – Provides structural data – Suitable for assessing folding/unfolding and ligand binding (T_m_ shift)	– High protein requirement – Relatively low throughput

Fluorescence polarization (FP) Peptide/compound displacement	384	Synergy 4 from BioTek ViewLux from PerkinElmer	Low μg/well	– High throughput – Identifies ligands that bind to a specific binding site – K_D_ determination for macromolecule interactions	– Need a labeled ligand – Indirect measurement of binding – Sensitive to compound fluorescence

⁎ (Alexandrov et al. 2008).

⁎⁎ Test runs by the SGC lab.
